# Association of Medicaid Expansion in Arkansas With Postpartum Coverage, Outpatient Care, and Racial Disparities

**DOI:** 10.1001/jamahealthforum.2021.4167

**Published:** 2021-12-17

**Authors:** Maria W. Steenland, Ira B. Wilson, Kristen A. Matteson, Amal N. Trivedi

**Affiliations:** 1 Population Studies and Training Center, Brown University, Providence, Rhode Island; 2 Department of Health Services, Policy, and Practice, Brown University School of Public Health, Providence, Rhode Island; 3 Department of Obstetrics and Gynecology, Women and Infants Hospital, Providence, Rhode Island; 4 Warren Alpert Medical School of Brown University, Providence, Rhode Island; 5 Providence VA Medical Center, Providence, Rhode Island

## Abstract

**Importance:**

Non-Hispanic Black individuals are disproportionally covered by Medicaid during pregnancy and, compared with non-Hispanic White individuals, have higher rates of postpartum coverage loss and mortality. Expanded Medicaid coverage under the Affordable Care Act may have increased continuity of coverage and access to care in the critical postpartum period in expansion states.

**Objective:**

To examine the association of Medicaid expansion in Arkansas with continuous postpartum coverage, postpartum health care use, and change in racial disparities in the study outcomes.

**Design, Setting, and Participants:**

This cohort study with a difference-in-differences analysis compared persons with Medicaid and commercially financed childbirth, stratified by race, using Arkansas’ All-Payer Claims Database for persons with a childbirth between 2013 and 2015. Race and ethnicity from birth certificate data were classified as Hispanic, non-Hispanic Black (hereafter Black), non-Hispanic White (hereafter White), and other (including Asian, Native American or Alaska Native, and Pacific Islander) or unknown race. Data were analyzed between June 2020 and August 2021.

**Exposures:**

Medicaid-paid childbirth after January 1, 2014.

**Main Outcomes and Measures:**

Continuous health insurance coverage and the number of outpatient visits during the first 6 months postpartum.

**Results:**

A total of 60 990 childbirths (mean [SD] age of birthing person, 27 [5.3] years; 67% White, 22% Black, and 7% Hispanic) were included, among which 72.3% were paid for by Medicaid and 27.7% were paid for by a commercial payer. Medicaid expansion in Arkansas was associated with a 27.8 (95% CI, 26.1-29.5) percentage point increase in continuous insurance coverage and an increase in outpatient visits of 0.9 (95% CI, 0.7-1.1) during the first 6 months postpartum, representing relative increases of 54.9% and 75.0%, respectively. Racial disparities in postpartum coverage decreased from 6.3 (95% CI, 3.9-8.7) percentage points before expansion to −2.0 (95% CI, −2.8 to −1.2) percentage points after expansion. However, disparities in outpatient care between Black and White individuals persisted after Medicaid expansion (preexpansion difference, 0.4 [95% CI, 0.2-0.6] visits; postexpansion difference, 0.5 [95% CI, 0.4-0.6] visits).

**Conclusions and Relevance:**

In this cohort study with a difference-in-differences analysis of 60 990 childbirths, Medicaid expansion was associated with higher rates of postpartum coverage and outpatient visits and lower racial and ethnic disparities in postpartum coverage. However, disparities in outpatient visits between Black and White individuals were unchanged. Additional policy approaches are needed to reduce racial and ethnic disparities in postpartum care.

## Key Points

**Question:**

Was Arkansas’ Medicaid expansion associated with an increase in postpartum insurance coverage and visits, and did racial disparities in these outcomes change after expansion?

**Findings:**

In this cohort study with difference-in-differences analysis of 60 990 childbirths, Medicaid expansion was associated with increased continuous postpartum insurance coverage and outpatient visits. While disparities between non-Hispanic Black and White individuals in continuous postpartum coverage decreased after expansion, racial disparities in postpartum outpatient visits were unchanged.

**Meaning:**

Medicaid expansion, and 12-month extensions of pregnancy Medicaid, may increase postpartum coverage and outpatient visits, but additional efforts are needed to reduce racial disparities in postpartum outpatient visits.

## Teaser

This cohort study examines the association of Medicaid expansion in Arkansas with continuous postpartum coverage, postpartum health care use, and change in racial disparities.

## Introduction

Maternal mortality in the US is higher than that of other high-income countries and is driven by large and persistent racial disparities.^1,2^ Compared with non-Hispanic White women, rates of maternal death are more than twice as high among American Indian and Alaska Native women and more than 3 times as high among non-Hispanic Black women.^1^ Because more than 50% of maternal deaths occur after childbirth, policy makers have focused on expanding health care coverage during the postpartum period.^3,4^ In 2013, average Medicaid income eligibility for pregnant people was 185% of the federal poverty level (FPL) but ranged from 133% to 300% of the FPL.^5^ However, in 2013, people who qualified for Medicaid owing to pregnancy lost coverage 60 days following delivery.^3^ Before the Affordable Care Act, approximately 26% of new mothers with income below the poverty level were uninsured in the year after childbirth.^6^ Postpartum insurance gaps are much higher among persons covered by Medicaid compared with persons covered by a commercial payer.^7^

By increasing income eligibility to 138% of the FPL for all adults, the Affordable Care Act’s Medicaid expansion increased the continuity of insurance coverage after childbirth among women with low income.^6,8^ In the years after expansion (2015-2018), among people who had prenatal Medicaid coverage, 10% were uninsured postpartum in expansion states compared with 36% in nonexpansion states.^9^

Between 2019 and 2020, the 116th US Congress considered 4 separate federal acts that included a federal mandate to extend pregnancy Medicaid coverage for 1 year postpartum.^4^ The 2021 American Rescue Plan included a new option that allows states to extend postpartum Medicaid coverage from 60 days to 1 year postpartum through a state plan amendment.^10^ Several states have proposed Section 1115 waivers or used state funds to expand coverage to more limited populations and services.^4^ Efforts to increase postpartum insurance coverage for people with Medicaid might have an especially large effect on Black women, because compared with non-Hispanic White individuals, non-Hispanic Black individuals are more likely to have low-income and Medicaid insurance and lose postpartum coverage.^11^

Existing evidence from individual states has shown that Medicaid expansion increased postpartum health care use in Colorado and Ohio^12,13^ and postpartum contraceptive use nationally.^14^ Medicaid expansion reduced maternal mortality,^15^ particularly among Black women. However, it is not known whether Medicaid expansion differentially increased health care use among Black women, the population with the highest rates of maternal morbidity and mortality.

In this cohort study, we add to this literature by focusing on the results of Arkansas’ Medicaid expansion. Arkansas expanded Medicaid coverage under the Affordable Care Act on January 1, 2014, through a “private option,” which enrolled newly eligible beneficiaries in Marketplace plans. Medicaid expansion in Arkansas increased income eligibility for parents from 16% of the FPL in 2013 to 138% in 2014 and from 0% to 138%, respectively, for nondisabled adults.^5^ This study compared changes in postpartum insurance and postpartum care after Medicaid expansion among persons covered by Medicaid during childbirth with concurrent changes among persons with commercial coverage. We also assessed changes in racial disparities in these outcomes before and after the state’s Medicaid expansion.

## Methods

### Study Design

We conducted a quasi-experimental difference-in-differences study that examined changes in insurance coverage and postpartum outpatient visits among adults (≥19 years of age) with Medicaid-financed births before (January through June 2013) and after expansion (January 2014 through December 2015) compared with concurrent trends among postpartum adults with commercially financed births. Postpartum people with Medicaid-financed births were classified as treated because, beginning January 1, 2014, they could qualify for extended Medicaid coverage beyond 60 days postpartum if their income was below 138% of the FPL, whereas before 2014, Medicaid postpartum coverage ended after 60 days and then reverted to much more restrictive parental income eligibility (ie, <17% of the FPL). People with Medicaid coverage during pregnancy must meet Medicaid’s pregnancy eligibility threshold of less than 200% of the FPL in Arkansas^16^ and are, therefore, likely to meet income eligibility requirements for adult Medicaid coverage after expansion.

We followed the Strengthening the Reporting of Observational Studies in Epidemiology (STROBE) reporting guideline.^17^ The data used in this study did not include identifiers and was therefore considered not human subject research by Brown University’s Human Research Protection Program.

### Data and Study Population

This study used Arkansas birth certificate records from 2013 through 2015 linked to medical claims from Arkansas’ All-Payer Claim Database (APCD) (2013-2016). This linkage was completed using last name and date of birth, which uniquely identifies approximately 96% of individuals giving birth in a given year.^18^ Therefore, the database included all claims during the 6 months after childbirth irrespective of whether the patient switched from Medicaid to commercial coverage, or from one commercial payer to another. Individuals were assigned to the treatment group (Medicaid or commercially financed childbirth) based on the payer associated with their insurance enrollment at the time of birth.

The unit of analysis in this study was a childbirth (n = 60 990). The study sample included 62% of registered births to adult Arkansas residents (19-50 years of age) during the study period after excluding births that were not covered by insurance (4.0%), those who did not match to an enrollment record at the time of birth in the APCD (26.0%), and those who matched but were covered by both Medicaid and a commercial payer at the time of childbirth (8.6%). See eFigure 1 in the Supplement for more detail on the sample construction and eTable 1 in the Supplement for a comparison of matched and nonmatched individuals.

### Outcomes

The primary outcomes were: (1) continuous insurance coverage during the 6 months after childbirth and (2) the number of outpatient visits, excluding emergency department (ED) visits, in the 6 months after childbirth. Any claim with a place of service code for an office or outpatient hospital was considered a non-ED outpatient visit. Grouped claims from the same person on the same day from the same health care professional were defined as a visit. Because Medicaid pregnancy coverage ends at 60 days postpartum, we also assessed the number of visits between day 1 and day 60 postpartum and between 61 days and 6 months postpartum.

### Exposure Variables and Covariates

The primary exposure was an interaction between Medicaid coverage at delivery and whether the delivery occurred after January 1, 2014. Covariates included age, race and ethnicity, and education level as self-reported on the birth certificate. Self-reported race and ethnicity from birth certificate data were classified as Hispanic, non-Hispanic Black (hereafter Black), non-Hispanic White (hereafter White), and other or unknown race. Racial groups in the other category included Asian, Native American or Alaska Native, and Pacific Islander. Age was categorized into the following groups: 19-24, 25-30, 31-34, and 35-50 years of age. Education was collapsed into a dichotomous indicator of completion of a college degree.

### Statistical Analysis

#### Regression Analysis

The main specification used multivariable linear regression models for each of the study outcomes. Each regression included an indicator variable for whether the birth was paid for by Medicaid or a commercial payer, an indicator for whether the birth took place after the start of Medicaid expansion in Arkansas, and an interaction term between Medicaid financing and the postexpansion indicator. Births between January 1, 2013, and June 30, 2013, were included in the preexpansion period. Given that the 6-month postpartum period for people with births between July 1 and December 31, 2013, overlapped with the preexpansion and postexpansion periods, this period was designated a *transitional period*, and those in 2014 and 2015 were classified as the postexpansion period. Regression models adjusted for age, education level, race and ethnicity, and county of residence with standard errors clustered at the individual level (9.2% of persons in the sample had more than 1 birth during the study period).

#### Racial and Ethnic Subgroup Analysis

We examined whether the association between expansion and the study outcomes was different among Black compared with White birthing people by conducting stratified difference-in-differences analysis and comparing the difference-in-differences coefficients between groups. The sample size of Hispanic individuals covered by a commercial payer was too small to conduct stratified difference-in-differences analysis in this group; however, we describe visual trends and interrupted time-series analysis among Hispanic persons with a Medicaid birth (eAppendix in the Supplement). Finally, we conducted a pre-post analysis to examine the change in racial disparities in the study outcomes by comparing differences in outcomes between White and Black postpartum people before and after Medicaid expansion in the full study population (ie, pooling together Black and White individuals).

#### Tests for Parallel Pretrends and Compositional Changes

Although the study data set only included 6 months of births that were fully unexposed to the policy, we tested whether the prepolicy trends in all of the study outcomes were parallel (eTable 2 in the Supplement). To test for changes in the composition of persons with Medicaid or commercial-financed births, we conducted the same difference-in-differences analysis described above, where the outcome variables were sample characteristics for each group, including total number of births, race and ethnicity, and average age. Analyses were conducted using Stata statistical software, version 17 (StataCorp). Statistical significance was set at *P* < .05, and all tests were 2-sided.

## Results

### Characteristics of the Study Population

The study included a total of 60 990 childbirths: 72.3% were paid for by Medicaid, and 27.7% were paid for by a commercial payer. Relative to persons with commercial childbirth coverage, persons with Medicaid coverage during childbirth were more likely to be in younger age groups (19-24 years old), were more likely to identify as Black and Hispanic, and were less likely to identify as White. Persons with Medicaid coverage were much less likely to have obtained college or higher-level education (5.1%) compared with persons with commercial coverage (55.5%) at childbirth (Table 1). Medicaid expansion was not associated with a change in the total births paid for by each payer nor with large changes in the racial and ethnic composition of the study groups (eTable 2 in the Supplement).

**Table 1.** Characteristics of Persons Giving Birth in Arkansas by Source of Insurance Coverage at Delivery, 2013-2015^a^

Characteristic	% (95% CI)	
	Medicaid (n = 44 103)	Commercial (n = 17 254)
Mean age at delivery, y	25.5 (25.5-25.6)	29.4 (29.3-29.5)
Age group, y		
19-24	50.0 (49.5-50.5)	16.0 (15.5-16.5)
25-30	32.8 (32.4-33.3)	44.5 (43.8-45.3)
31-35	10.7 (10.4-11.0)	24.8 (24.1-25.4)
36-50	6.5 (6.2-6.7)	14.7 (14.2-15.2)
Race and ethnicity		
Hispanic	10.8 (10.5-11.1)	3.0 (2.8-3.3)
Non-Hispanic Black	27.6 (27.1-28.0)	7.1 (6.7-7.5)
Non-Hispanic White	58.0 (57.5-58.5)	86.2 (85.7-86.7)
Other or unknown race^b^	3.7 (3.5-3.9)	3.7 (3.4-4.0)
Education level		
Less than college	94.9 (94.7-95.1)	44.5 (43.7-45.2)
College or higher	5.1 (4.9-5.3)	55.5 (54.8-56.3)
Cesarean birth	33.5 (33.1-34.0)	32.9 (32.2-33.7)

^a^ Data are analyzed from the Arkansas All-Payer Claims Database.

^b^ Racial groups in the other category included Asian, Native American or Alaska Native, and Pacific Islander.

### Postpartum Coverage

**Figure 1.**
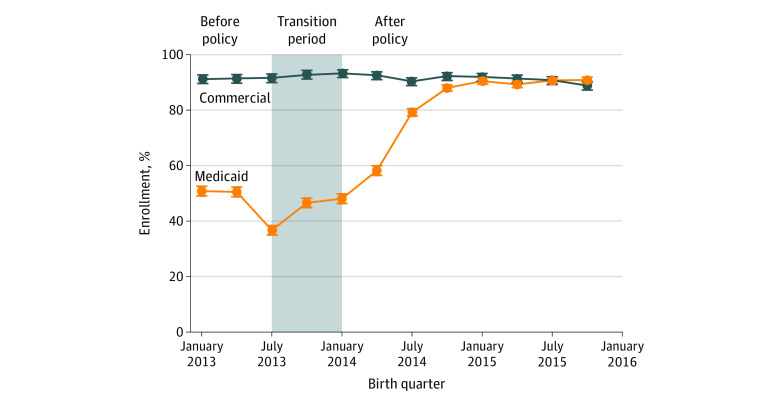
Proportion of Persons With 6 Months of Continuous Insurance Coverage After Childbirth by Source of Coverage at Childbirth, 2013-2015 Data are analyzed from the Arkansas All-Payer Claims Database. Each plotted point represents the percentage of persons delivering in that quarter who were continuously enrolled in health insurance coverage during the entire 6 months after childbirth. The shaded area represents the transition period, designated as such because the 6-month postpartum period for persons who gave birth between July and December 2013 overlapped only partially with the Medicaid expansion period.

Figure 1 plots the proportion of persons with 6 months of continuous postpartum insurance coverage among persons with Medicaid and commercial coverage at childbirth between January 2013 and December 2015. Before Medicaid expansion, the trend in continuous coverage among persons with Medicaid coverage did not differ statistically from the trend among persons with commercial coverage (eTable 3 in the Supplement). Before Medicaid expansion, between January and June 2013, approximately 91.3% (95% CI, 90.2%-92.4%) of persons with commercial coverage at childbirth had continuous coverage during the 6 months after childbirth. The share of continuous coverage in this group remained stable during 2014 and 2015 (Figure 1 and Table 2). Among persons covered by Medicaid at childbirth, before Medicaid expansion, 50.6% (95% CI, 49.5%-51.8%) had continuous coverage during the 6 months postpartum (Figure 1 and Table 2). The share of persons with Medicaid childbirth coverage who were continuously covered for 6 months postpartum increased to 69.3% in 2014 and 90.0% in 2015 (Table 2). Medicaid expansion was associated with a 27.8 (95% CI, 26.1-29.5) percentage point increase in continuous 6 to 12 month postpartum coverage (Table 2).

**Table 2.** Changes in Continuous Enrollment and Number of Outpatient Visits During the First 6 Months Postpartum Associated With Medicaid Expansion, 2013-2015^a^

Variable	Medicaid financed				Commercial financed				Difference-in-differences	
	% (95% CI)		Unadjusted difference between preexpansion and postexpansion, percentage point (95% CI) [n = 36 434]	*P* value	% (95% CI)		Unadjusted difference between preexpansion and postexpansion, percentage point (95% CI) [n = 13 930]	*P* value	Adjusted difference-in-differences, percentage point (95% CI)	*P* value
	Preexpansion (Jan-Jun 2013) [n = 6856]	Postexpansion (2014-2015) [n = 29 578]			Preexpansion (Jan-Jun 2013) [n = 2723]	Postexpansion (2014-2015) [n = 11 207]				
**Continuous insurance enrollment during first 6 mo after delivery**										
Enrollment	50.6 (49.5 to 51.8)	80.0 (79.5 to 80.4)	29.3 (28.0 to 30.6)	<.001	91.3 (90.2 to 92.4)	91.8 (91.3 to 92.3)	0.5 (−0.6 to 1.7)	.36	27.8 (26.1 to 29.5)	<.001
**No. of outpatient visits during first 6 mo after delivery**										
Full 6 mo postpartum	1.2 (1.1 to 1.3)	2.0 (2.0 to 2.0)	0.8 (0.7 to 0.9)	<.001	3.2 (3.0 to 3.3)	3.0 (2.9 to 3.1)	−0.2 (−0.4 to 0.0)	.09	0.9 (0.7 to 1.1)	<.001
Days 1 through 60	0.5 (0.5 to 0.6)	0.7 (0.7 to 0.7)	0.2 (0.1 to 0.2)	<.001	1.2 (1.1 to 1.3)	1.1 (1.1 to 1.1)	−0.1 (−0.2 to 0.0)	.06	0.2 (0.1 to 0.3)	<.001
Day 61 through 6 mo	0.6 (0.6 to 0.7)	1.3 (1.2 to 1.3)	0.6 (0.6 to 0.7)	<.001	2.0 (1.9 to 2.1)	1.9 (1.8 to 2.0)	−0.1 (−0.2 to 0.1)	.23	0.7 (0.5 to 0.9)	<.001

^a^ Data are analyzed from the Arkansas All-Payer Claims Database. Unadjusted differences are calculated using *z* tests for differences in continuous coverage and *t* tests for differences in outpatient visits. Difference-in-differences regression models adjusted for age, education level, race and ethnicity, and county of residence. Standard errors are clustered at the individual level.

### Use of Outpatient Care Postpartum

**Figure 2.**
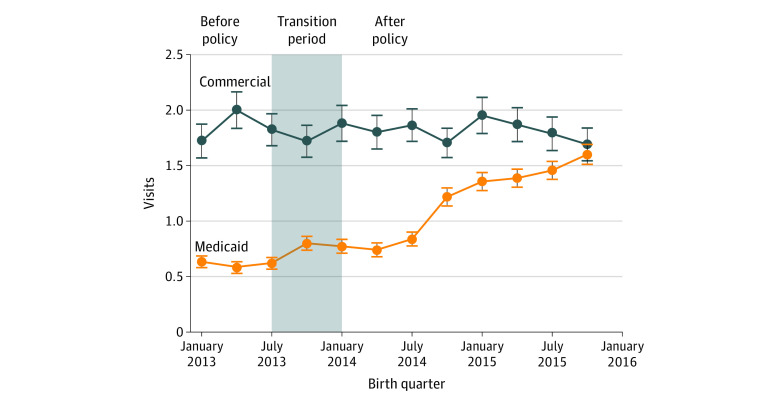
Mean Number of Non–Emergency Department Outpatient Visits Between 61 Days and 6 Months Postpartum Among Persons With Medicaid and Commercially Financed Childbirth, 2013-2015 Data are analyzed from the Arkansas All-Payer Claims Database. Each plotted point represents the average number of non–emergency department outpatient visits during the 6 months after childbirth among persons delivering in that quarter. The shaded area represents the transition period, designated as such because the 6-month postpartum period for persons who gave birth between July and December 2013 overlapped only partially with the Medicaid expansion period.

Figure 2 plots the mean number of non-ED outpatient visits between 61 days and 6 months postpartum among persons with Medicaid and commercial coverage at childbirth; trends in non-ED outpatient visits in the first 60 days and the full 6 months postpartum are presented in eFigures 2 and 3 in the Supplement. Before Medicaid expansion, the trend in all 3 outpatient visit outcomes among persons with Medicaid coverage was statistically different from the trend among persons with commercial coverage; however, the difference is no longer statistically significant after including the transition period in the analysis (eTable 3 in the Supplement). Comparing the change between groups during the entire 6-month postpartum period, Medicaid expansion was associated with a 0.9 (95% CI, 0.7-1.1) visit increase, or a relative increase of 75.0% compared with the visit rate of 1.2 visits within the first 6 months postpartum in the preexpansion period. This increase was driven primarily by an increase in visits between 61 days and 6 months postpartum (Table 2).

### Subgroup Analysis

Among persons with Medicaid-covered childbirth, continuous postpartum coverage was lower among Hispanic individuals than non-Hispanic White and Black individuals, as was the number of outpatient visits within the first 6 months postpartum (eFigures 4 and 5 in the Supplement). Medicaid expansion was associated with a similarly large increase in continuous postpartum coverage among Hispanic persons compared with non-Hispanic White and Black individuals but a smaller increase in postpartum outpatient visits relative to other racial and ethnic groups (eTable 4 in the Supplement).

Before Medicaid expansion among persons covered by Medicaid at childbirth, Black individuals had slightly higher rates of 6-month continuous postpartum coverage (58.6% [95% CI, 56.4%-60.9%]) compared with White individuals (53.7% [95% CI, 52.2%-55.3%]) (Table 3). Medicaid expansion was associated with a similarly large increase in coverage in both groups (27.8 [95% CI, 25.7-29.8] percentage points for White individuals and 28.1 [95% CI, 23.1-33.0] percentage points for Black individuals). Before Medicaid expansion among persons covered by Medicaid, Black birthing people had an average of 1.4 (95% CI, 1.3-1.6) visits, and White birthing people had an average of 1.3 (95% CI, 1.2-1.3) visits in the first 6 months postpartum. In the first 6 months postpartum, Medicaid expansion was associated with an increase in visits of 1.14 (95% CI, 0.90-1.38) among White birthing people and 0.91 (95% CI, 0.21-1.61) among Black birthing people (Table 3).

**Table 3.** Changes in Continuous Enrollment and Number of Outpatient Visits During the First 6 Months Postpartum Associated With Medicaid Expansion Stratified by Race, 2013-2015^a^

Variable	Medicaid			*P* value, unadjusted difference between preexpansion and postexpansion	Commercial insurance			*P* value, unadjusted difference between preexpansion and postexpansion	Difference-in-differences (95% CI) [n = 43 792]	*P* value
	% (95% CI)		Difference, percentage point (95% CI) [n = 31 133]		% (95% CI)		Difference, percentage point (95% CI) [n = 12 918]			
	Jan-Jun 2013 [n = 5775]	2014 and after [n = 25 358]			Jan-Jun 2013 [n = 2531]	2014 and after [n = 10 387]				
**Continuous insurance enrollment during first 6 mo postpartum**										
Black birthing people	58.6 (56.4 to 60.9)	87.5 (86.7 to 88.2)	28.8 (27.0 to 30.7)	<.001	91.6 (90.5 to 92.7)	92.2 (91.7 to 92.8)	0.6 (−0.6 to 1.8)	.33	28.1 (23.1 to 33.0)	<.001
White birthing people	53.7 (52.2 to 55.3)	82.4 (81.8 to 83.0)	28.6 (27.2 to 30.1)	<.001	91.7 (87.8 to 95.6)	92.2 (90.3 to 94.1)	0.5 (−3.7 to 4.8)	.81	27.8 (25.7 to 29.8)	<.001
**No. of outpatient visits during first 6 mo postpartum**										
Black birthing people	1.4 (1.3 to 1.6)	2.0 (1.9 to 2.0)	0.5 (0.4 to 0.7)	<.001	3.3 (2.7 to 3.9)	2.9 (2.6 to 3.3)	−0.4 (−1.1 to 0.4)	.37	0.91 (0.21 to 1.61)	.01
White birthing people	1.3 (1.2 to 1.3)	2.2 (2.2 to 2.3)	1.0 (0.8 to 1.1)	<.001	3.2 (3.0 to 3.4)	3.0 (3.0 to 3.1)	−0.2 (−0.4 to 0.0)	.11	1.14 (0.90 to 1.38)	<.001

^a^ Data are analyzed from the Arkansas All-Payer Claims Database. Unadjusted differences are calculated using *z* tests for differences in continuous coverage and *t* tests for differences in outpatient visits. Difference-in-differences regression models adjusted for age, education level, race and ethnicity, and county of residence. Standard errors are clustered at the individual level.

### Pre-Post Comparison of Racial Disparities Between Black and White Individuals

Before Medicaid expansion, a smaller percentage of Black birthing individuals had continuous 6-month insurance coverage after childbirth (61.7% [95% CI, 59.6%-63.8%]) compared with White individuals (68.0% [95% CI, 66.8%-69.1%]) (eTable 5 in the Supplement). In the 2 postexpansion years, the percentage of Black and White birthing people with continuous 6-month postpartum coverage increased to 87.9% (95% CI, 87.2%-88.6%) and 85.9% (95% CI, 85.5%-86.3%), respectively (eTable 5 in the Supplement). Before expansion among all individuals in the study population, White individuals had an average of 2.0 (95% CI, 1.9-2.1) visits in the first 6 months postpartum compared with 1.6 (95% CI, 1.4-1.7) visits among Black individuals. Although there was no difference in postpartum insurance coverage between Black and White individuals in the postexpansion years, racial disparities in the number of visits in the first 6 months postpartum between Black and White individuals remained after Medicaid expansion (2.5 [95% CI, 2.5-2.6] visits among White individuals and 2.0 [95% CI, 2.0-2.1] visits among Black individuals).

## Discussion

Results of this study showed that Medicaid expansion was associated with an increase in continuous 6-month postpartum insurance coverage among individuals with Medicaid coverage during childbirth, nearly eliminating the substantial preexpansion differences in continuous enrollment by childbirth payer. We also found that Medicaid expansion was associated with an increase in the number of non-ED outpatient postpartum visits during the first 6 months postpartum among persons with Medicaid coverage at childbirth. The majority of the increase in visits during the 6 months after childbirth occurred from 61 days through 6 months postpartum. This finding is consistent with the hypothesis that Medicaid expansion would primarily increase visits after the end of Medicaid pregnancy coverage at 60 days postpartum.

This study also contributes new evidence comparing the association between Medicaid expansion and racial disparities in postpartum coverage and health care use. We found that Medicaid expansion was associated with similarly large increases in postpartum coverage and visits during the first 6 months after childbirth among Black and White individuals. We also evaluated changes in racial disparities in the study outcomes after expansion. In a pre-post analysis combining persons with Medicaid and commercially paid childbirth, we found that after Medicaid expansion, the population-level racial disparity in continuous 6-month postpartum coverage was eliminated but racial disparities in postpartum outpatient visits persisted.

Medicaid expansion in Arkansas increased income eligibility from 16% to 138% of the FPL. We found larger associations of the expansion in Arkansas with coverage and utilization than those documented in studies that focused on Ohio and Colorado, 2 states with more generous preexpansion Medicaid eligibility.^12,13^ We also added evidence on the association between expansion and the study outcomes by race comparing the difference-in-differences estimates between Black and White individuals, a question of considerable public health and policy importance given the large racial disparities in maternal morbidity and mortality.

These findings have several important policy implications. First, the findings suggest that lack of insurance coverage after childbirth was an important driver of differences in health care utilization between persons with commercial and Medicaid-paid childbirth. Arkansas’ low preexpansion income eligibility for parental Medicaid coverage (16% of the FPL) makes the state comparable to the 12 remaining nonexpansion states, which had an average parental income eligibility of 41% of the FPL in 2019 and were disproportionately located in the South. Therefore, Medicaid expansion has the potential to be an effective policy tool to increase postpartum coverage and outpatient postpartum health care among low-income postpartum individuals in states that have not yet expanded Medicaid. Pregnancy Medicaid extensions, which have been passed or implemented in several states with additional pending proposals in several other states, are likely to accomplish similar gains in coverage and care in states with low postpartum coverage.

Racial disparities in postpartum outpatient care persisted after Medicaid expansion in Arkansas. Therefore, the present findings suggest that increased coverage may not be sufficient to reduce racial disparities in outpatient postpartum care, and additional policy efforts, particularly those focused on addressing institutional and interpersonal racism,^19,20^ are required. For instance, previous research has found that Black women experience high levels of patient-reported discrimination during childbirth hospitalizations^21^ and that postpartum pain is less well managed among Hispanic and non-Hispanic Black women compared with non-Hispanic White women.^22^ Furthermore, there is evidence that geographic access to health care is more limited in majority Black neighborhoods.^23^ These additional barriers may account for ongoing differences in non-ED outpatient care between non-Hispanic Black and White women. Legislative efforts, such as the Momnibus Act of 2021,^24^ which target a broad range of factors, including social determinants, the perinatal workforce, and health care quality improvement, are likely to be needed to improve maternal health equity.

### Limitations

This study has several limitations. While postpartum persons can be tracked between payers in the study data set, the Arkansas APCD does not include self-pay outpatient care. Therefore, we may have underestimated the number of outpatient postpartum visits, particularly before Medicaid expansion when coverage levels were very low. Additionally, the Arkansas APCD does not include enrollees in self-insured plans, and the present findings would be biased if the change in the trends in coverage and visits in this group differed from the trends in the employer-sponsored and marketplace commercially insured groups. Third, it is possible that these findings could be biased by changes in the composition of births paid for by Medicaid and commercial payers after Medicaid expansion. However, we show that Medicaid expansion was not associated with a change in the total births paid for by each payer and was not associated with large changes in the racial and ethnic composition of the study groups. A fourth limitation is that, because the Arkansas APCD began in 2013, the study data set only included 6 months of childbirths whose 6-month postpartum period did not overlap at all with Medicaid expansion. We found statistically different pretrends between groups for the visit outcomes, but this difference is no longer statistically significant when we add the transition period, before coverage had increased, to the regression analysis. Six months of data, particularly in the commercially insured group, which had a smaller sample size, was not sufficient to identify stable prepolicy trends in the study outcomes. Fifth, while all people with Medicaid coverage at childbirth were included in the treatment group, those with income between 138% and 200% of the FPL would not have been eligible for parental Medicaid, and this classification may have biased the results toward the null. Sixth, the study outcomes did not include a health outcome, and outpatient visits may not improve health outcomes. Finally, the data did not contain a measure of prepregnancy health status, which could have caused bias in the results if it changed differentially between groups over time.

## Conclusions

In this cohort study with difference-in-differences analysis of 60 990 childbirths, Medicaid expansion nearly closed the gap in continuous 6-month postpartum insurance coverage and non-ED outpatient visits in the first 6 months postpartum between persons with Medicaid and commercially paid childbirth. While Medicaid expansion increased postpartum coverage and outpatient visits among non-Hispanic Black individuals, disparities in postpartum outpatient care between non-Hispanic Black and non-Hispanic White individuals persisted after Medicaid expansion, suggesting the need for additional interventions beyond coverage to improve access to postpartum care among non-Hispanic Black individuals.

## Notes

Supplement. **eFigure 1.** Sample inclusion flow-chart **eTable 1.** Characteristics of persons giving birth in Arkansas from 2013 to 2015, by whether the birth matched to an enrollment record in the Arkansas All-Payer Claims Database at the time of birth **eTable 2.** Changes in the characteristics of persons with Medicaid and commercially paid childbirth associated with Medicaid expansion, 2013-2015 **eAppendix.** Tests for parallel pre trends and robustness checks **eTable 3.** Pre-period monthly trend Differences between persons with Medicaid and commercial coverage during childbirth **eTable 4.** Results from interrupted time series regression among persons with Medicaid coverage, 2013-2015 **eFigure 2.** Mean number of non-ED outpatient visits in the first 60 days postpartum visits among persons with Medicaid and commercially financed childbirth, 2013-2015 **eFigure 3.** Mean number of outpatient visits during the first six months postpartum among persons with Medicaid and commercially financed childbirth, 2013-2015 **eFigure 4.** Proportion of persons with Medicaid-covered childbirth who had six months of continuous insurance coverage after childbirth, by race/ethnicity, 2013-2015 **eFigure 5.** Mean number of non-ED outpatient visits during the first 6 months postpartum among persons with Medicaid financed childbirth, by race/ethnicity, 2013-2015 **eTable 5.** Racial disparities in continuous enrollment and number of outpatient visits during the first 6 months postpartum, 2013-2015
eReference
