# Supplementary material for: Association of Medicaid Expansion in Arkansas With Postpartum Coverage, Outpatient Care, and Racial Disparities
Source: JAMA Health Forum. 2021 Dec 17;2(12):e214167. doi: 10.1001/jamahealthforum.2021.4167 (PMC8796925; doi:10.1001/jamahealthforum.2021.4167)
Supplement: Supplement. — eFigure 1. Sample inclusion flow-chart eTable 1. Characteristics of persons giving birth in Arkansas from 2013 to 2015, by whether the birth matched to an enrollment record in the Arkansas All-Payer Claims Database at the time of birth eTable 2. Changes in the characteristics of persons with Medicaid and commercially paid childbirth associated with Medicaid expansion, 2013-2015 eAppendix. Tests for parallel pre trends and robustness checks eTable 3. Pre-period monthly trend Differences between persons with Medicaid and commercial coverage during childbirth eTable 4. Results from interrupted time series regression among persons with Medicaid coverage, 2013-2015 eFigure 2. Mean number of non-ED outpatient visits in the first 60 days postpartum visits among persons with Medicaid and commercially financed childbirth, 2013-2015 eFigure 3. Mean number of outpatient visits during the first six months postpartum among persons with Medicaid and commercially financed childbirth, 2013-2015 eFigure 4. Proportion of persons with Medicaid-covered childbirth who had six months of continuous insurance coverage after childbirth, by race/ethnicity, 2013-2015 eFigure 5. Mean number of non-ED outpatient visits during the first 6 months postpartum among persons with Medicaid financed childbirth, by race/ethnicity, 2013-2015 eTable 5. Racial disparities in continuous enrollment and number of outpatient visits during the first 6 months postpartum, 2013-2015 eReference [file jamahealthforum-e214167-s001.pdf]

Supplemental Online Content

Steenland MW, Wilson IB, Matteson KA, Trivedi AN. Association of Medicaid expansion in Arkansas with postpartum coverage, outpatient care, and racial disparities. *JAMA Health Forum*. 2021;2(12):e214167. doi:10.1001/jamahealthforum.2021.4167

eFigure 1. Sample inclusion flow-chart
eTable 1. Characteristics of persons giving birth in Arkansas from 2013 to 2015, by whether the birth matched to an enrollment record in the Arkansas All-Payer Claims Database at the time of birth
eTable 2. Changes in the characteristics of persons with Medicaid and commercially paid childbirth associated with Medicaid expansion, 2013-2015
eAppendix. Tests for parallel pre trends and robustness checks
eTable 3. Pre-period monthly trend Differences between persons with Medicaid and commercial coverage during childbirth
eTable 4. Results from interrupted time series regression among persons with Medicaid coverage, 2013-2015
eFigure 2. Mean number of non-ED outpatient visits in the first 60 days postpartum visits among persons with Medicaid and commercially financed childbirth, 2013-2015
eFigure 3. Mean number of outpatient visits during the first six months postpartum among persons with Medicaid and commercially financed childbirth, 2013-2015
eFigure 4. Proportion of persons with Medicaid-covered childbirth who had six months of continuous insurance coverage after childbirth, by race/ethnicity, 2013-2015
eFigure 5. Mean number of non-ED outpatient visits during the first 6 months postpartum among persons with Medicaid financed childbirth, by race/ethnicity, 2013-2015
eTable 5. Racial disparities in continuous enrollment and number of outpatient visits during the first 6 months postpartum, 2013-2015
eReference

This supplemental material has been provided by the authors to give readers additional information about their work.

© 2021 Steenland MW et al. *JAMA Health Forum.*

Study population:
Twenty six percent of births to insured adult Arkansas residents did not match to an enrollment record because they were covered in self-insured plans, which are not required to submit data to the APCD, or due to limitations of matching birth certificates to enrollment based on name and date of birth (i.e., name changes and typographical errors).

eFigure 1: Sample inclusion flow-chart

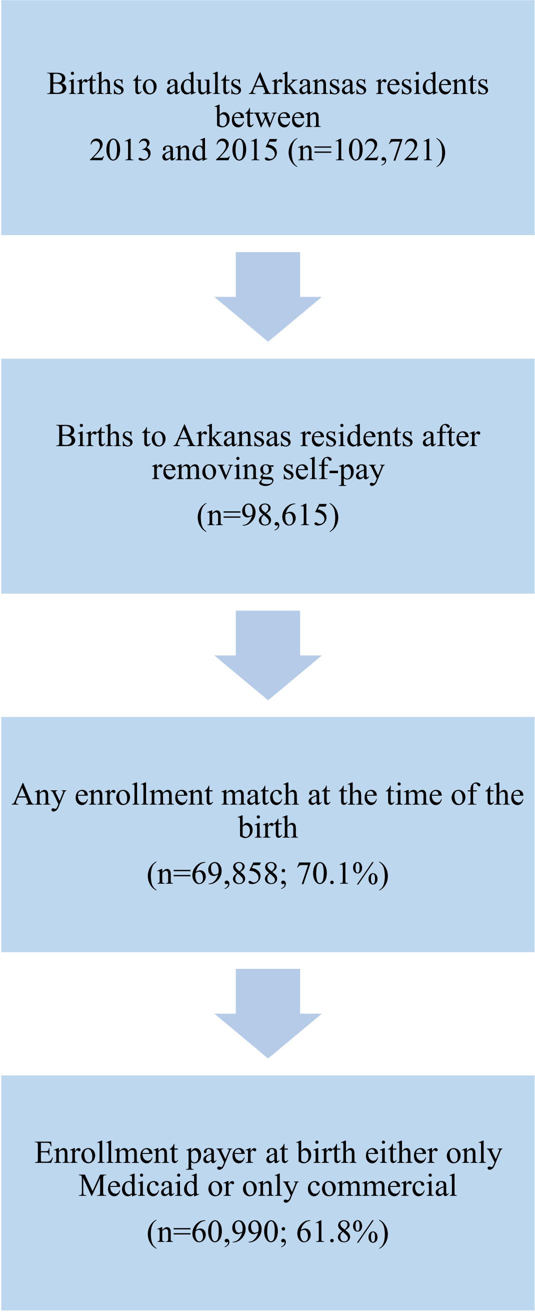

© 2021 Steenland MW et al. *JAMA Health Forum.*

eTable 1: Characteristics of persons giving birth in Arkansas from 2013 to 2015, by whether the birth matched to an enrollment record in the Arkansas All-Payer Claims Database at the time of birth

Variable	Matched (n=60,990)	Unmatched (n=37,599)
	Percent (95% CI)	Percent (95% CI)
Mean age in years	26.6 (26.6, 26.7)	27.7 (27.6, 27.7)
Age 19-24	40.4 (40.0, 40.8)	32.5 (32.0, 32.9)
Age 25-30	36.1 (35.7, 36.5)	38.1 (37.6, 38.6)
Age 31-35	14.7 (14.4, 14.9)	17.8 (17.5, 18.2)
Age 36-50	8.8 (8.6, 9.1)	11.6 (11.3, 11.9)
Hispanic	8.6 (8.4, 8.8)	11.9 (11.6, 12.2)
Non-Hispanic black	21.8 (21.5, 22.2)	12.4 (12.1, 12.8)
Non-Hispanic white	65.8 (65.5, 66.2)	70.4 (69.9, 70.8)
Hispanic	8.6 (8.4, 8.8)	11.9 (11.6, 12.2)
Non-Hispanic other or unknown race	3.7 (3.5, 3.8)	5.3 (5.1, 5.5)
Less than college	80.6 (80.2, 80.9)	71.8 (71.3, 72.2)
College or greater	19.4 (19.1, 19.8)	28.2 (27.8, 28.7)
Cesarean birth	26.6 (26.6, 26.7)	27.7 (27.6, 27.7)

Authors' analysis of the Arkansas All Payer Claims Database, 2013–15. Table includes only adults (19 year or older) who were residents of Arkansas at the time of birth.

© 2021 Steenland MW et al. *JAMA Health Forum.*

eTable 2: Changes in the characteristics of persons with Medicaid and commercially paid childbirth associated with Medicaid expansion, 2013-2015

	Medicaid January-June 2013	Medicaid 2014 and after	Medicaid Difference	Unadjusted difference between pre-and post-expansion	Commercial January-June 2013	Commercial 2014 and after	Commercial Difference	Unadjusted difference between pre-and post-expansion	Difference-in-Differences	
	Percent (95% CI)	Percent (95% CI)	Percentage Points (95% CI)	p-value	Percent (95% CI)	Percent (95% CI)	Percentage Points (95% CI)	p-value	Percentage Points (95% CI)	p-value
Age	25.3 (25.2, 25.4)	25.6 (25.6, 25.7)	0.3 (0.2, 0.5)	0.000	29.3 (29.2, 29.5)	29.6 (29.5, 29.6)	0.2 (0.0, 0.4)	0.034	0.1 (-0.1, 0.3)	0.373
Hispanic	12.2 (11.5, 13.0)	10.1 (9.8, 10.4)	-2.1 (-2.9, -1.3)	0.000	3.0 (2.3, 3.6)	3.1 (2.8, 3.4)	0.1 (-0.6, 0.8)	0.785	-2.0*** (-3.1, -1.0)	0.000
Black	27.6 (26.5, 28.6)	26.8 (26.3, 27.3)	-0.7 (-1.9, 0.4)	0.213	7.1 (6.1, 8.1)	6.8 (6.3, 7.3)	-0.3 (-1.4, 0.8)	0.574	-0.5 (-2.0, 0.9)	0.477
White	56.8 (55.6, 58.0)	59.2 (58.7, 59.8)	2.5 (1.2, 3.7)	0.000	86.0 (84.7, 87.3)	86.2 (85.5, 86.8)	0.2 (-1.2, 1.6)	0.787	2.1* (0.3, 3.9)	0.024
Total births	1,145 (1,144, 1,146)	1,214 (1,213, 1,215)	69 (66, 72)	0.000	455 (454, 456)	485 (484, 485)	29 (28, 31)	0.000	0.03 (-0.06, 0.12)	0.485

SOURCE Authors' analysis of the Arkansas All Payer Claims Database, 2013–15. NOTES Difference-in-differences regression models adjusted for age, education, race and county of residence. Standard errors clustered at the individual level. *p < 0.05 **p < 0.01 ***p < 0.001

© 2021 Steenland MW et al. *JAMA Health Forum.*

eAppendix. Tests for parallel pre trends and robustness checks

We tested for differential trends in continuous postpartum coverage and number of visits in the first six months postpartum by childbirth payer. We fail to reject the null that the trends in 6-month continuous coverage and mean visits during the first 60 days postpartum were parallel. However, we find evidence of non-parallel pre-trends for the number of visits between 61 days and 6 months postpartum (β=-0.1, 95% CI: [-0.1, -0.0]), and for total visits within the first six months postpartum (β=-0.1, 95% CI: [-0.1, -0.0]).

However, we do not believe that the finding of non-parallel trends for two of the study outcomes is a likely cause of bias in this study. While tests of parallel trends are common in published difference-in-differences literature, recent methodologic studies have pointed out that while parallel pre-trends provides suggestive evidence of counterfactual parallel trends, it is neither necessary nor sufficient for the parallel counterfactual trends assumption to hold.[1]

As our study includes only six months of fully pre-policy data, we have limited information on which to conduct baseline tests for parallel pre-trends. Due to the limited number of pre-policy months, and the high variance of the trend for visits among persons with commercially paid childbirth, we find implausibly large estimates for the pre-trend in this group that is not robust to inclusion of subsequent months in the study period. The pre-policy trend for postpartum visits between days 61 months and 6 years and the full six months postpartum among persons with a commercially paid birth was a 0.1 visit increase per month (95% CI 0.0, 0.1). Extrapolating this trend to the end of the study would have resulted in an increase of 3.5 visits for both outcomes, or almost a 300% increase from baseline for visits between 61 days and six months postpartum and a 220% increase from baseline for visits during the full six months postpartum. Further, extending months included in the analysis to include the transition period completely eliminated this increasing trend and difference in the pre-trends. These findings suggest that six months of pre-policy data is not sufficient to reliably identify pre-policy trends among commercially paid births.

© 2021 Steenland MW et al. *JAMA Health Forum.*

eTable 3: Pre-period monthly trend Differences between persons with Medicaid and commercial coverage during childbirth

	Continuous 6-month Postpartum enrollment	Visits in the first 60 days postpartum	Visits between 61 days and 6 months postpartum	Visits during the Full six months postpartum
January – June 2013	Coefficient (95% CI)	Coefficient (95% CI)	Coefficient (95% CI)	Coefficient (95% CI)
Medicaid-paid birth	-39.3*** (-43.1, -35.4)	-0.5*** (-0.7, -0.4)	-0.8*** (-1.0, -0.6)	-0.7*** (-0.9, -0.5)
Trend	0.4 (-0.4, 1.2)	0.0 (-0.0, 0.1)	0.1* (0.0, 0.1)	0.0* (0.0, 0.1)
Medicaid-paid birth*trend	-0.2 (-1.1, 0.7)	-0.0* (-0.1, -0.0)	-0.1*** (-0.1, -0.0)	-0.1*** (-0.1, -0.0)
Constant	97.7*** (87.7, 107.6)	0.1 (-0.6, 0.8)	0.7** (0.2, 1.3)	1.1*** (0.6, 1.7)
All months 2013				
Medicaid-paid birth	-38.0*** (-40.5, -35.5)	-0.6*** (-0.7, -0.4)	-1.2*** (-1.5, -1.0)	-1.8*** (-2.1, -1.5)
Trend	0.7*** (0.4, 1.0)	-0.0 (-0.0, 0.0)	0.0 (-0.0, 0.0)	0.0 (-0.0, 0.0)
Medicaid-paid birth*trend	-1.1*** (-1.4, -0.8)	-0.0 (-0.0, 0.0)	0.0 (-0.0, 0.0)	0.0 (-0.0, 0.1)
Constant	86.6*** (79.3, 93.8)	1.1*** (0.8, 1.4)	1.8*** (1.3, 2.3)	2.9*** (2.3, 3.6)

*p < 0.05 **p < 0.01 ***p < 0.001

© 2021 Steenland MW et al. *JAMA Health Forum.*

eTable 4: Results from interrupted time series regression among persons with Medicaid coverage, 2013-2015

	Continuous enrollment	Full six months postpartum	Days 1 through 60	Day 61 and 6 months
All birthing persons	Percentage Points (95% CI)	Number (95% CI)	Number (95% CI)	Number (95% CI)
Quarterly trend before Medicaid expansion	1.95*** (1.28, 2.61)	-0.02 (-0.06, 0.02)	-0.01 (-0.02, 0.01)	-0.02 (-0.05, 0.01)
Level change immediately after Medicaid expansion	-10.90* (-21.35, -0.46)	0.03 (-0.39, 0.45)	-0.04 (-0.17, 0.09)	0.07 (-0.29, 0.44)
Change in quarterly trend after Medicaid expansion	7.09*** (5.29, 8.89)	0.15** (0.07, 0.24)	0.06*** (0.04, 0.08)	0.09* (0.02, 0.17)
Non-Hispanic Black birthing persons				
Quarterly trend before Medicaid expansion	1.38* (0.36, 2.40)	-0.02 (-0.08, 0.03)	-0.01 (-0.02, 0.00)	-0.02 (-0.04, 0.00)
Level change immediately after Medicaid expansion	-8.74 (-20.28, 2.81)	0.16 (-0.24, 0.57)	-0.01 (-0.09, 0.06)	0.18 (-0.19, 0.54)
Change in quarterly trend after Medicaid expansion	8.04*** (6.61, 9.46)	0.21*** (0.13, 0.29)	0.08*** (0.06, 0.09)	0.14*** (0.08, 0.19)
Non-Hispanic white birthing persons				
Quarterly trend before Medicaid expansion	1.52 (-0.57, 3.61)	-0.09* (-0.17, -0.01)	-0.03 (-0.06, 0.00)	-0.06 (-0.12, 0.00)
Level change immediately after Medicaid expansion	-7.36 (-16.43, 1.71)	0.24 (-0.19, 0.67)	0.00 (-0.12, 0.13)	0.24 (-0.09, 0.57)
Change in quarterly trend after Medicaid expansion	6.83*** (4.19, 9.48)	0.19** (0.08, 0.30)	0.08*** (0.04, 0.12)	0.11** (0.03, 0.19)
Hispanic birthing persons				
Quarterly trend before Medicaid expansion	-0.26 (-2.18, 1.67)	-0.06 (-0.15, 0.02)	-0.02* (-0.04, -0.00)	-0.04* (-0.08, -0.01)
Level change immediately after Medicaid expansion	-1.11 (-9.33, 7.10)	0.30 (-0.28, 0.87)	0.05 (-0.05, 0.15)	0.24* (0.03, 0.46)
Change in quarterly trend after Medicaid expansion	6.99*** (4.54, 9.45)	0.04 (-0.11, 0.20)	0.04* (0.01, 0.07)	0.01 (-0.08, 0.10)

Authors' analysis of the Arkansas All Payer Claims Database, 2013–15. Interrupted time series regression models adjusted for age, education, and race. Standard errors adjusted for autocorrelation. Quadratic term used to account for nonlinear trend in the outcome after the start of Medicaid expansion.
*p < 0.05 **p < 0.01 ***p < 0.001

© 2021 Steenland MW et al. *JAMA Health Forum.*

eFigure 2: Mean number of non-ED outpatient visits in the first 60 days postpartum visits among persons with Medicaid and commercially financed childbirth, 2013-2015

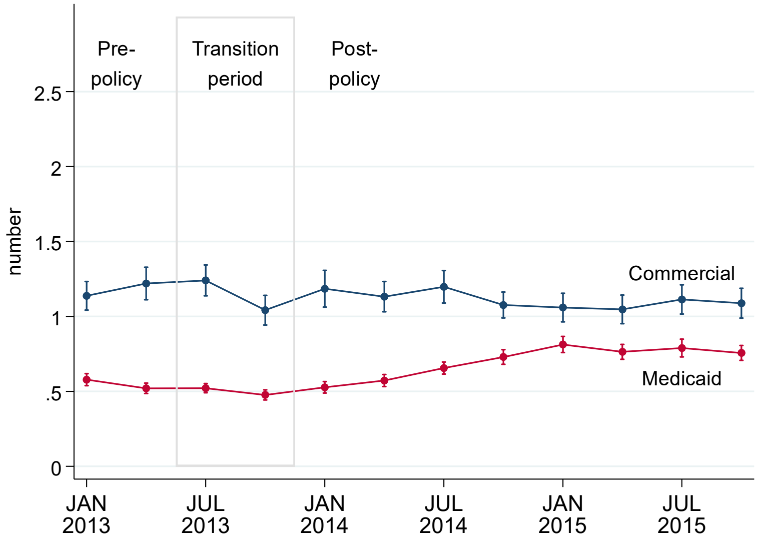

eFigure 3: Mean number of outpatient visits during the first six months postpartum among persons with Medicaid and commercially financed childbirth, 2013-2015

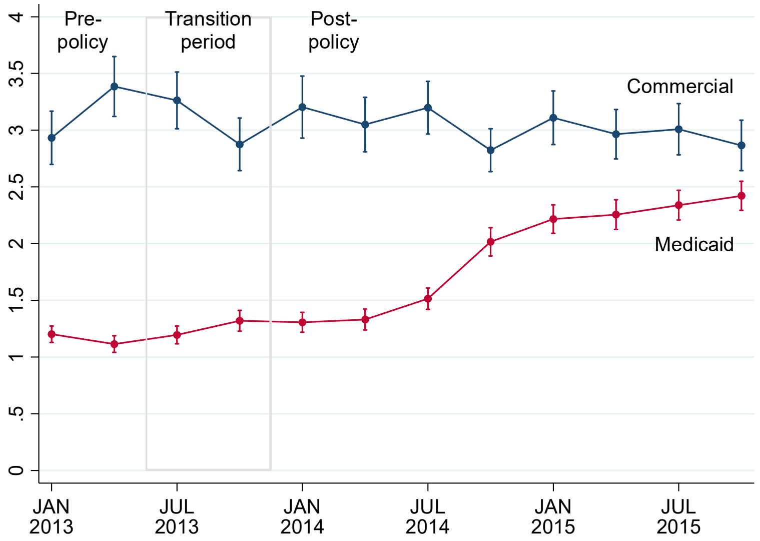

© 2021 Steenland MW et al. *JAMA Health Forum.*

eFigure 4: Proportion of persons with Medicaid-covered childbirth who had six months of continuous insurance coverage after childbirth, by race/ethnicity, 2013-2015

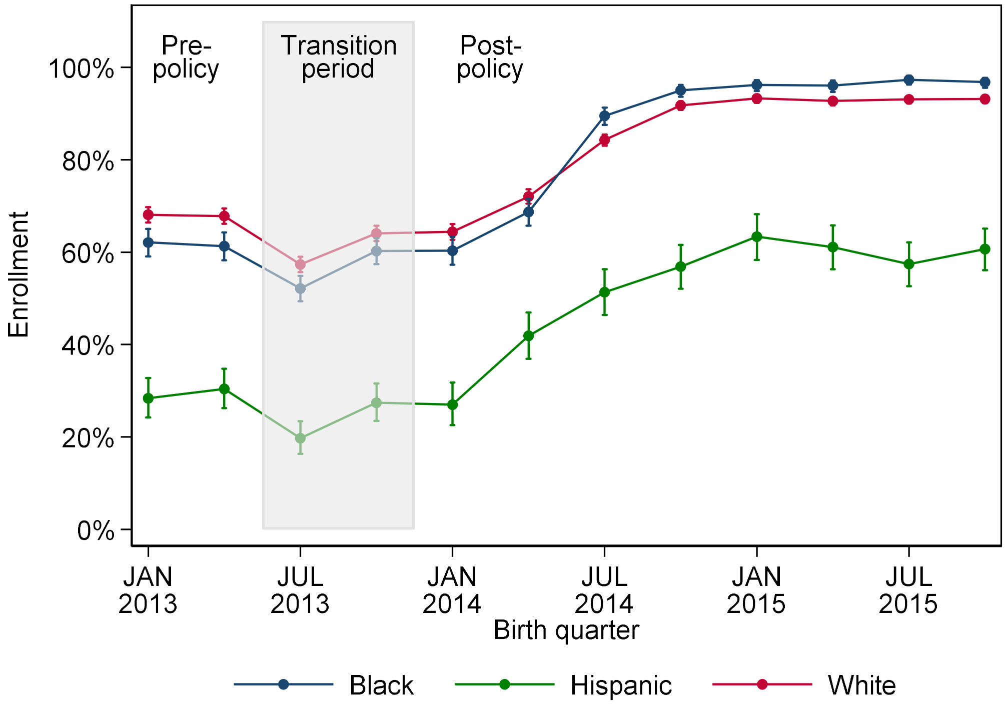

eFigure 5: Mean number of non-ED outpatient visits during the first 6 months postpartum among persons with Medicaid financed childbirth, by race/ethnicity, 2013-2015

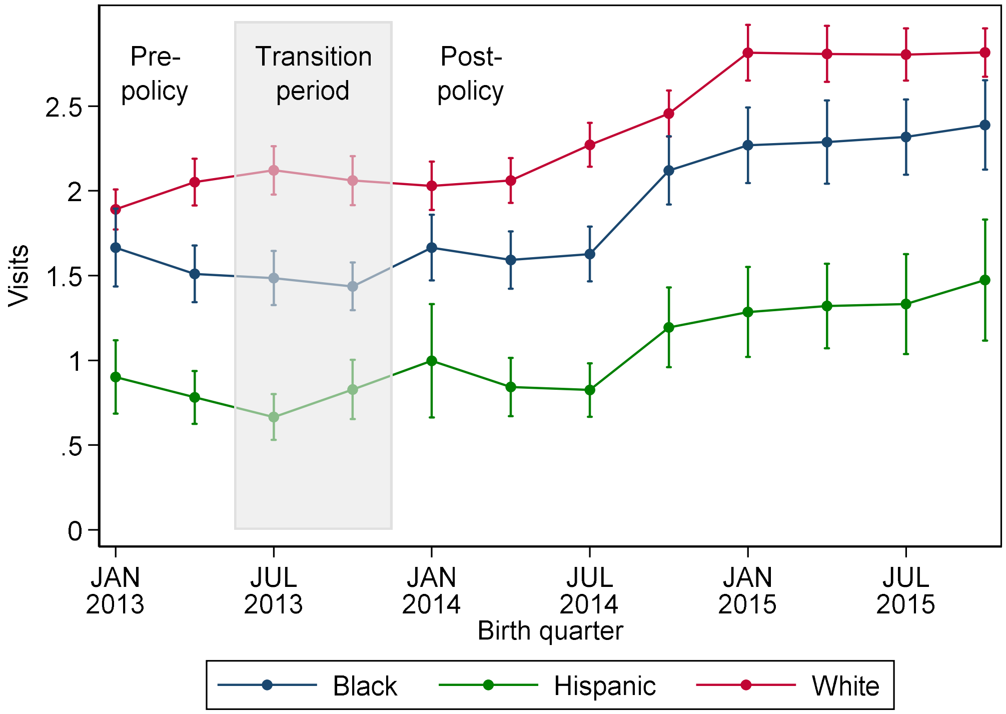

© 2021 Steenland MW et al. *JAMA Health Forum.*

eTable 5: Racial disparities in continuous enrollment and number of outpatient visits during the first 6 months postpartum, 2013-2015

	Pre-expansion<br>January-June 2013<br>Percent (95% CI)	Post-expansion<br>(2014/2015)<br>Percent (95% CI)
Continuous enrollment		
Black birthing people	62 (59.6, 63.8)	88 (87.2, 88.6)
White birthing people	68 (66.8, 69.1)	86 (85.5,86.3)
Number of outpatient visits		
Black birthing people	1.6 (1.4, 1.7)	2.0 (2.0,2.1)
White birthing people	2.0 (1.9,2.1)	2.5 (2.5,2.6)

© 2021 Steenland MW et al. *JAMA Health Forum.*

eReference

1. Kahn-Lang A, Lang K. The promise and pitfalls of differences-in-differences: Reflections on 16 and pregnant and other applications. *Journal of Business & Economic Statistics*. 2020;38(3):613-620.

© 2021 Steenland MW et al. *JAMA Health Forum.*
